# Templating of Monomeric Alpha-Synuclein Induces Inflammation and SNpc Dopamine Neuron Death in a Genetic Mouse Model of Synucleinopathy

**DOI:** 10.21203/rs.3.rs-5269499/v1

**Published:** 2024-11-20

**Authors:** Matthew D. Byrne, Peyman Petramfar, Jae-Kyung Lee, Richard Jay Smeyne

**Affiliations:** Thomas Jefferson University; Thomas Jefferson University; University of Georgia; Thomas Jefferson University

**Keywords:** a-Synuclein, microglia, SNpc, preformed filaments, Lewy Bodies, neuroinflammation

## Abstract

While the etiology of most cases of Parkinson’s disease (PD) are idiopathic, it has been estimated that 5–10% of PD arise from known genetic mutations. The first mutations described that leads to the development of an autosomal dominant form of PD are in the SNCA gene that codes for the protein alpha-synuclein (α-syn). α-syn is an abundant presynaptic protein that is natively disordered and whose function is still unclear. In PD, α-syn misfolds into multimeric b-pleated sheets that aggregate in neurons (Lewy Bodies/neurites) and spread throughout the neuraxis in a pattern that aligns with disease progression. Here, using IHC, HC, HPLC, and cytokine analysis, we examined the sequelae of intraparenchymal brain seeding of pre-formed fibrils (PFFs) and monomeric α-syn in C57BL/6J (WT) and A53T SNCA mutant mice. We found that injection of PFFs, but not monomeric α-syn, into the striatum of C57BL/6J mice induced spread of aggregated α-syn, loss of SNpc DA neurons and increased neuroinflammation. However, in A53T SNCA mice, we found that both PFFs and monomeric α-syn induced this pathology. This suggests that the conformation changes in α-syn seen in the A53T strain can recruit wild-type α-syn to a pathological misfolded conformation which may provide a mechanism for the induction of PD in humans with SNCA duplication/triplication.

## Introduction

Parkinson’s disease (PD) is a multisystem neurodegenerative disorder affecting over six million people worldwide ^1^. While the majority of PD cases are considered to be idiopathic, a number of well characterized genetic mutations have been identified that account for approximately 5–10% of those diagnosed with PD. Interestingly, the presentation of genetic and sporadic cases are often indistinguishable ^2^, including both the characteristic motor and non-motor symptoms. The classic motor symptoms of PD include bradykinesia, tremor, and rigidity, while the non-motor symptoms include gut dysfunction, sleep disorders, speech pathologies and psychiatric issues such as depression and hallucinations ^1,3^. The onset of both the motor and non-motor symptoms is often insidious, but symptoms are progressive. Currently, there are no disease-modifying therapies available for patients ^4^. In the central nervous system (CNS), PD pathology is characterized by the progressive loss of the dopaminergic (DA) neurons located in the substantia nigra pars compacta (SNpc) and degeneration of their terminals in the striatum that ultimately result in loss of striatal dopamine and induction of neuroinflammation manifested in astrocytosis, microgliosis and increased pro-inflammatory cytokine levels ^5,6^. However, the most characteristic feature of parkinsonian CNS pathology is the presence of dense proteinaceous intraneuronal inclusions, called Lewy bodies, that are localized within neurons in the brain. Although many proteins are present in these inclusions, the most abundant of these proteins is alpha synuclein (α-Syn)^7^. The gene encoding α-Syn (SNCA) was first linked to PD by the discovery of a kindred family who displayed an accelerated onset of PD symptoms with a dominant pattern of inheritance, oft times at significantly earlier ages that the traditional age of PD symptom onset ^8^. Subsequent studies have discovered additional families with mutations in this gene, including those with point mutations, duplications, and triplications of the SNCA gene; all of which are causative for PD. Additional studies have also demonstrated an association between α-Syn levels and the risk of developing sporadic PD ^9^.

While the normal function of α-Syn is still poorly understood, SNCA encodes a 140 amino acid protein that is enriched at the presynaptic terminal, and accounts for as much as 1% of all proteins in the cytosol of cells in the CNS^10^. Under homeostatic conditions, α-Syn exists as a disordered monomer, however, it is prone to aggregating into higher order soluble oligomeric species as well as insoluble aggregates, characterized by b-pleated sheets and phosphorylation at serine129 (p129Ser) ^11^. These insoluble aggregates are toxic both *in vitro* and *in vivo*, however, their toxicity can depend on both the conformational and molecular weight of the assembly^12^.

While the pathological hallmark of PD is the presence of Lewy bodies in the SNpc DA neurons, there is a predictable progression of α-Syn pathology, characterized by the progressive appearance of aggregated misfolded α-Syn in the neuroaxis that coincides with the clinical progression of the disease ^13^. Temporally, this Lewy pathology first appears in the peripheral neurons of the gut followed by appearance in the dorsal motor nucleus of the vagus nerve (DMV) and olfactory bulb. Later, pathology is noted in the lower brainstem nuclei, the SNpc, and then finally, in some cases, to neocortical nuclei pathological burden ^13^. The pattern of α-Syn misfolding, along direct circuits, suggests that α-Syn pathology is transmissible ^14^. This observation was further confirmed from studies that found that transplanted fetal DA neurons, when implanted into the brains of PD patients developed this Lewy pathology despite a significant age difference in the neurons ^15^.

While it is hypothesized that the spread and accumulation of α-Syn aggregates in neurons drives neurodegeneration in PD, recent studies have suggested that there is a significant contribution from neuroinflammation that can both create and/or trigger a pathogenic cellular environment that is permissive for α-Syn aggregation ^16^. For example, it has been shown using in vitro models that α-Syn activates microglia which can synergize neurotoxicity ^17^. It has also been shown that this process can initiate and perpetuate a toxic cycle where continued release of aggregated α-Syn from damaged neurons induces activation of microglia and astrocytes which then leads to subsequent neurodegeneration ^17^. While this link between neuronal cell death, α-Syn aggregation, and glial activation has been studied in PD, there is still much debate about the initiating event(s) of PD pathology and role of inflammation.

Since α-Syn has been confirmed to play a central pathogenic role in several well-known synucleinopathies ^18^, powerful models of disease have been generated that allow for the experimental interrogation of the mechanisms of synuclein pathogenesis. These include the development of several mouse models that contain known pathogenic mutations (A53T, A30P) in the SNCA gene ^19,20^. However, for the most part, none of these mouse models have been able to faithfully recreate the nigrostriatal degeneration and α-Syn aggregation present in humans with PD ^21^. To overcome this issue, an in vivo model for PD was developed that specifically focused on the role of misfolded, aggregated α-Syn. Here, monomeric recombinant human α-Syn is incubated to generate insoluble aggregated preformed fibrils (PFFs). Sonication can break up larger fibrils into smaller fibrillar species that are small enough to be taken into neurons ^22^. Using this model, a single intracerebral inoculation with α-Syn PFF’s has been shown to induce a pathology that approximates that seen in PD including insoluble aggregates of α-Syn in neurons, synaptic dysfunction, dysregulation of striatal dopamine release, and even nigrostriatal degeneration ^22 23^. The mechanism for this appears to be that these PFFs act as a “seed”, capable of triggering aggregation of non-misfolded endogenous α-Syn in cells ^24^.

In this study we examined the neuropathological consequences of intracerebral inoculation with full length human α-Syn PFFs or α-Syn monomers injected in C57BL/6J wildtype mice or C57BL/6J mice harboring a double P1-derived artificial chromosome (PAC) transgene overexpressing the human A53T mutation on a mouse SNCA−/− background^20^. We chose to use human α-Syn to optimize molecular and biological compatibility with the A53T mice, aiming to characterize the pathological and importantly neuroinflammation hallmarks ^25^. We hypothesized that the transgenic line overexpressing α-Syn would result in accelerated pathology compared to wild type animals but did not expect monomeric α-Syn to induce pathology in either mouse species. We found that injection of PFFs into the striatum or hippocampus of both C57BL/6J WT and A53T transgenic mice resulted in a predictable and progressive pattern of α-Syn pathology that was circuit specific. Additionally, we observed that injection of the monomeric forms of α-Syn into C57BL/6J mice did not induce any progressive synuclein pathology. However, when the monomeric form of α-Syn was intracerebrally injected into mice harboring the human A53T mutation, we observed the induction of a synucleinopathy that was indistinguishable from that seen following injection of PFFs. This suggests that dysregulated overexpression of α-Syn, when in sufficient quantity, can induce misfolding of normal synuclein. We hypothesize that this may be the reason why humans carrying the A53T or even overexpression (duplication or triplication of the wild-type SNCA gene) develops PD pathology at a significantly earlier time than seen in idiopathic or even other familial forms of Parkinson’s disease.

## Materials and Methods

### Animals

All animals used in this study were purchased from the Jackson Labs (Bar Harbor ME). C57BL/6J mice (Cat # 00064) or FVB;129S6-Tg(SNCA*A53T)1Nbm-*Snca*^*tm1Nbm*^Tg(SNCA*A53T)2Nbm/J (Also known as: dbl-PAC-tg(SNCAA53T);Snca−/−) (A53T, Cat 010799); the latter backcrossed to C57BL/6J for > 10 generations. It is also important to note that the A53T transgenic mice are on a mouse SNCA −/− background, meaning that all synuclein expressed in these animals are of the human species. All mice were 6–12 weeks old when PFFs were intracerebrally injected. All of the experimental procedures in the animals were performed in accordance with the NIH Guide for the Care and Use of Laboratory Animals and all protocols were approved by the Thomas Jefferson University IACUC (Protocol 1892).

### Preparation of α-Syn PFFs and Quality Control

Recombinant human α-Syn expressed in E. Coli was obtained from Proteos (Kalamazoo, MI). The monomeric form of α-Syn was centrifuged at 4°C for 10 minutes at 15,000 x g. After centrifugation, the supernatant was removed and its protein concentration was and determined using a Nanodrop Model 2000 spectrophotometer (Fisher Scientific). For each sample the monomer was diluted to a final concentration of 5mg/ml in ~ 100mM NaCl, ~ 7.5mM Tris, and ~ 10mM phosphate and adjusted to pH 7.4. As 100ug aliquot was placed into an orbital shaker at 37°C for 7 days at 1,000 RPM to induce fibrillization. Successful fibrillization in confirmed via transmission electron microscopy and sedimentation assay, with greater amounts of protein in the pellet versus supernatant fraction (Suppl Fig. 1).

### Endotoxin Reporter Assay

The level of endotoxin in the synuclein preparations were evaluated via a HEK-Blue mTLR4 reporter assay. Commercially available α-Syn (Proteos) and various concentrations of LPS were used to measure TLR4 activation and subsequent NF-kB signaling. Proteos α-Syn was shown to not induce TLR4 activation (Suppl Fig. 2).

### Preparation of Surgical Material

**Figure 1:**
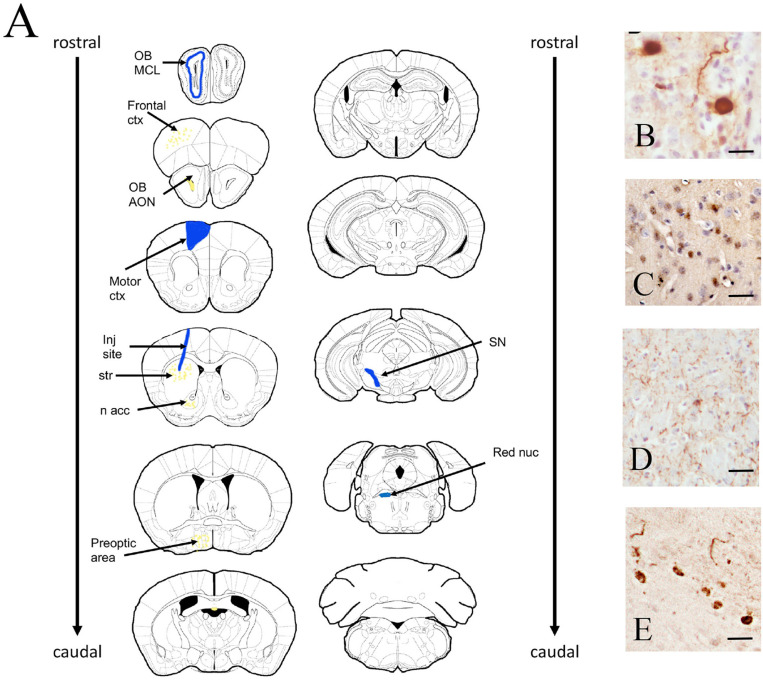
Map of pSer-129 immunopositive neurons in C57BL/6J and A53T mice following intrastriatal injection of PFFs. A. 60 days after intrastriatal injection of PFFs into the dorsolateral striatum we observed (from rostral to caudal) pSer129 α-syn immunoreactive neurons throughout the neuraxis. Areas highlighted in blue showed medium to high levels of pSer129 α-syn, while areas in yellow showed low to medium expression. B. Photomicrograph of pSer129 α-syn in the AON. C) photomicrograph of pSer129 α-syn in the motor cortex, D. photomicrograph of pSer129 α-syn in the striatum around the injection site, E. photomicrograph of pSer129 α-syn in the SNpc. This is the appearance of cell used to quantitate pSer129 α-syn-positive DA neurons for quantitation. OB: olfactory bub, MCL: mitral cell layer of the olfactory bulb, AON: accessory olfactory nucleus, inj site: injection site, str: striatum, n. acc: nucleus accumbens, SN: substantia nigra pars compacta. Scale bar B= 10 um, C-E, 25 um.

PFFs or monomeric α-Syn were diluted to 2ug/ul in sterile 1X PBS. These samples were then sonicated using a microtip sonicator at power level 2 for .5 second ON/.5 second OFF pulses, with pausing every 10 seconds to prevent excess heat and frothing. Following sonication, successful disruption of the fibrils was confirmed using transmission electron microscopy, with average fibril being ~ 50nm min length (Fig. 1). Monomeric protein was centrifuged at 15,000xg at 4C before surgical session, with the supernatant retained for use in the surgical injections. Injections.

### Stereotaxic Injection of PFFS and monomeric α-Syn into Mouse Brain

Mice were anesthetized using a 3% mixture of isoflurane/oxygen. Following exposure of the skull, a small hole was drilled using a robotic stereotaxic instrument (Neurostar, Tubingen, Germany) at locations above the striatum or hippocampus. 2ul of diluted monomer or PFFs (2ug/ul) were injected into either the dorsal lateral striatum (AP 0.86, DV −2.5, ML 1.8) or rostral hippocampus (AP −2.18, DV −1.25, ML 1.75) using a 10ul Hamilton microsyringe at a constant rate of 0.4ul per minute. The syringe was left in place for 5 minutes after which it was slowly withdrawn. The scalp was then closed using proline suture and the wound was treated with topical lidocaine.

### Immunohistochemistry

30, 60, or 180 days post injection, mice were deeply anesthetizing with an intraperitoneal injection of 0.9% Avertin. After loss of corneal and deep tendon reflexes, mice were transcardially perfused with phosphate-buffered saline (PBS, pH 7.4), followed by 3% paraformaldehyde in PBS, pH 7.4. Brains were then dissected out of the skull following perfusion and post-fixed overnight in fresh 3% PFA. Brains were then dehydrated using a gradient solution of ethanol, defatted in xylenes, and then embedded in paraffin (Paraplas-Xtra, Fisher Scientific), and cut serially at 10um. Every section from the olfactory bulb to the anterior aspects of the cerebellar-midbrain junction was mounted to Superfrost-Plus slides (Fisher Scientific), five sections per slide. Every fourth slide (first series) was immunostained for p129-synuclein (1:40k, mouse monoclonal, 81a ab184674, abcam Cambridge, UK). The next slide in the series (second series) was immunolabeled for both tyrosine hydroxylase (TH) as a marker of dopaminergic neurons (1:250; mouse monoclonal, T1299, Sigma-Aldrich, MO, USA) and ionized calcium-binding adapter molecule 1 (Iba-1) (1:200; rabbit polyclonal, 019–19741, Wako, VA, USA). The double labeling was carried out using a two-color DAB/VIP protocol. All sections were counterstained with a Nissl stain (cresyl violet).

#### Qualitative Determination of p129-synuclein burden.

Sections from all brains in the anatomical studies, spaced 200 um apart were immunostained for pSer129 α-syn. Sections were examined under 20x and 40x objectives for the presence of insoluble aggregates of pSer129 α-syn. A survey of structures throughout the CNS (Suppl Table 1) was qualitatively scored for the relative presence of pSer129 α-syn by an observer blinded to the experimental conditions. Structures were scored a either low (0–20% of cells in the region containing pSer129 α-syn aggregates, medium (20–50% of cells in the region containing pSer129 α-syn aggregates) or high (greater than 50% of cells in the region containing pSer129 α-syn aggregates) based on the highest expression on any single section within that region. Once the scoring for each animal was complete, the animals were unblinded and the relative SYN burden was determined. This was done by examining the scores for each structure in each animal in the group. Animals scored as low were assigned an ordinate score of 0, medium were assigned an ordinate score of 2 and those scored as high were assigned an ordinate score of 4. For each group, the ordinate numbers were summed and divided by the total number of animals. This average number was used to determine a relative burden for each structure in each experimental group. In addition to the qualitative scoring, we also stereologically estimated the number of pSer129 α-syn immunopositive DA neurons within the SNpc by model-based stereology ^26^ using the physical disector (StereoInvestigator, MBF Bioscience).

#### Quantitative Assessement of SNpc DA neurons and microglia.

The total number of SNpc tyrosine hydroxylase (TH)-positive DA neurons (+ Nissl) were estimated by model-based stereology ^26^ using the physical disector (StereoInvestigator, MBF Bioscience). Total DA neurons were estimated from the entire rostral to caudal aspect of the SN. Microglia numbers were stereologically estimated using design-based stereology using the optical fractionator (StereoInvestigator, MBF Biosciences) ^27^. In addition to total number, we also categorized microglia as resting or active. Microglia were deemed as “resting” if the Iba-1-positive cell body was < 3 microns in diameter and also possessed long slender processes. Microglia were counted as activated when the cell body was > 3 microns but also had an irregular shape with shorter and thickened processes ^28,29^. Statistical analysis was performed using one way ANOVA followed by a priori individual comparisons (Tukey post hoc test)(Prism version 10.3, GraphPad Software.

### Determination of striatal catecholamine content

Mice were deeply anesthetized with Avertin and transcardially perfused with 0.9% saline to remove the majority of the blood from the brain vasculature. Brains were rapidly removed and placed in a brain matrix (Model BS-AL-5000C, Braintree Scientific, Braintree. MA) and sliced into 1 mm thick sections. Dissected sections were placed on an ice-cooled plate and tissue was dissected from the SN (Bregma: −2.70- −3.70), striatum (Bregma: +0.14-+1.26mm), brainstem (Bregma: −5.40- −6.70mm), cortex (Bregma: −1.70- −2.70mm) and the hippocampus (Bregma: −1.70- −2.70mm) were dissected ^30^ and rapidly frozen on dry ice until processed. Tissues were homogenized, using a handheld sonic tissue dismembrator, in 100–750 ul of 0.1M TCA containing 0.01M sodium acetate, 0.1mM EDTA, and 10.5% methanol (pH 3.8). Ten microliters of this homogenate was used for the protein assay. The samples were then spun in a microcentrifuge at 10,000 g for 20 minutes. Supernatant was removed for HPLC-electrochemical detection analysis. HPLC was performed using a Kinetix 2.6um C18 column (4.6 × 100 mm, Phenomenex, Torrance, CA USA). The same buffer used for tissue homogenization is used as the HPLC mobile phase.

For final determination of catecholamine content, the protein concentration in cell pellets was determined by BCA Protein Assay Kit (Thermo Scientific). Ten microliter tissue homogenates were distributed into 96-well plate and 200 ml of mixed BCA reagent (25 ml of Protein Reagent A is mixed with 500 μl of Protein Reagent B) was added. Plates were incubated at room temperature for two hours for the color development. A BSA standard curve is run at the same time. Absorbance is measured by the plate reader (POLARstar Omega), purchased from BMG LABTECH Company. Statistical analysis was performed using one way ANOVA followed by a priori individual comparisons (Tukey post hoc test) (Prism version 10.3, GraphPad Software.

### Cytokine Assay

To examine the expression of cytokine/chemokines, brain tissue, prepared as described for HPLC analysis, was mechanically homogenized in Bioplex cell lysis buffer containing factors 1 and 2 (Bio-Rad, CA, USA) and centrifuged at 4500xg. The total protein concentration of each sample was determined using the BCA assay (Pierce, IL, USA), with bovine serum albumin as a standard, per the manufacturer’s protocol. Individual vials of cytokine/chemokine beads were sonicated, vortexed and then mixed with assay buffer (Milliplex Map Kit, MCYTOMAG-70K, Millipore, MA, USA). Working standards were made by diluting the stock concentration (10,000 pg/ml) in assay buffer. Brain and serum samples were added in equal volumes (25ul) into the wells of a 96-well plate containing 25ul of assay buffer and 25ul of cytokines mixed beads and plate was incubated overnight at + 4C. Then the plate was washed and incubated with the following: (i) detection antibodies, (ii) Straptavidin-Phycoerythrin, (iii) sheath fluid. The plates were then run on a Luminex 200^™^ according to the manufacturer’s recommended procedures and the data analyzed using BioPlex Manager 4.1 software (Bio-Rad, Hercules, CA). All samples were run in duplicates with data expressed as pg/mg total protein. Statistical analysis was performed using one way ANOVA followed by a priori individual comparisons (Tukey)(Prism version 10.3, GraphPad Software.

## Results

### Qualitative Assessement of Propagation of a-syn pathology after intracerebral injection of Preformed Filaments of α-syn.

**Figure 2:**
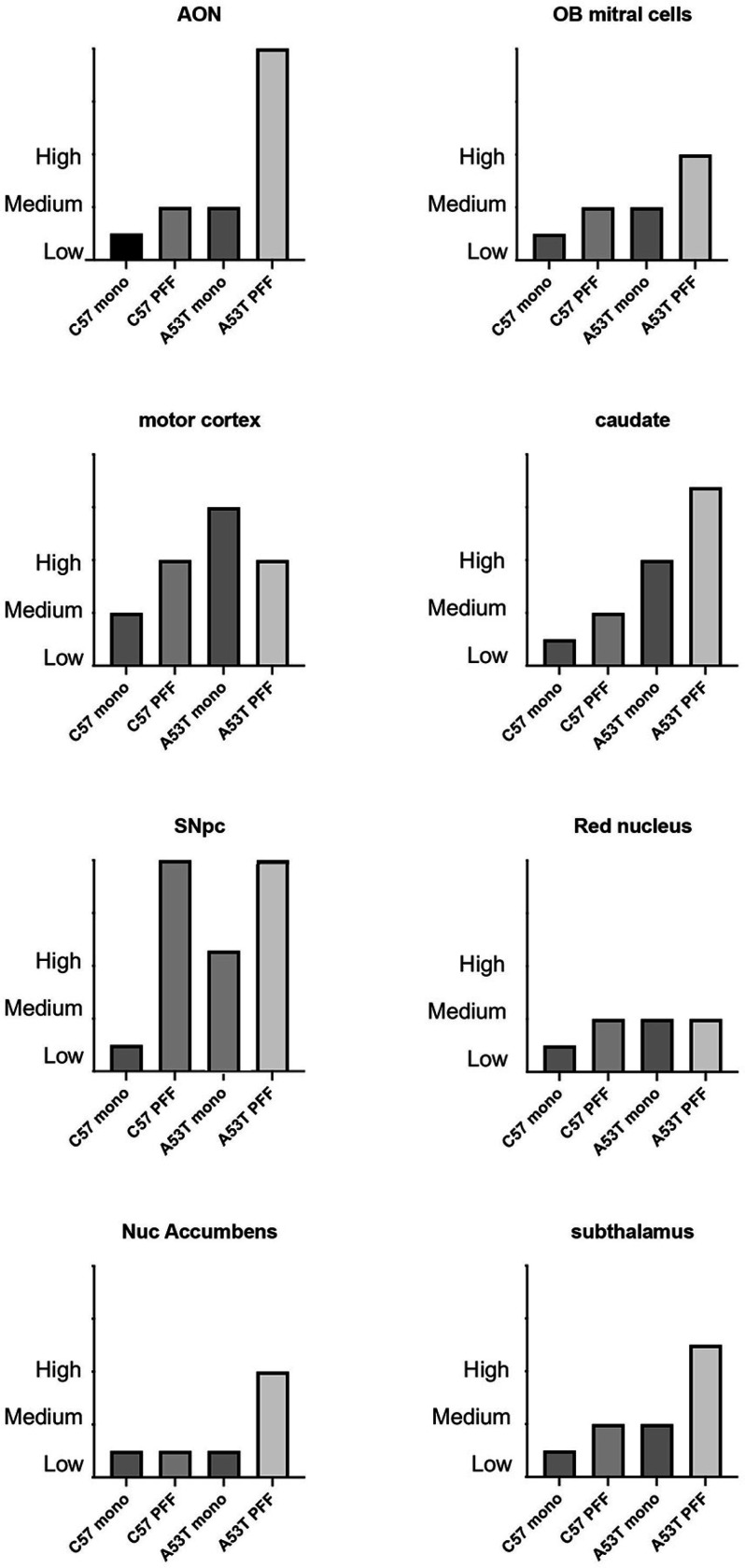


Following injections of PFF’s into the dorsal striatum of both C57BL/6J and A53T animals, we observed an ipsilateral distribution of pSer129 α-syn throughout the structures of the nigrostriatal pathway (Fig. 1). In C57BL/6J animals, pSer129 α-syn immunopositive neurons was first seen outside of the striatum at 30 days post injection and the expression was observed to be maximal at 60dpi. When pSer129 α-syn expression was examined at 180 dpi, we observed qualitatively lower levels of these immunopositive neurons than were seen at 60 days (Fig. 2).

To examine if the nigrostriatal pathway was at higher risk for pSer129 α-syn aggregation than other regions of the neuraxis, we injected a separate cohort of mice with PFFs into the rostral hippocampus. Like the striatal injections, we observed maximal transmission of insoluble pSer129 α-syn at 60 days, but the pattern of transmission was to structures that were primarily associated with the limbic system (Suppl Fig. 3). In C57BL/6J mice that were injected with monomeric α-syn, in both striatum as well as hippocampus, we did not observe any pSer129 α-levels of pSer129 α-syn-immunopositive neurons were seen in the A53T mice, the pattern of pSer129 α-syn was identical to that observed in the C57BL/6J mice. However, unlike the C57BL/6J mice, when A53T mice were injected with monomeric α-syn into the striatum or hippocampus, we observed pSer129 α-syn immunopositive neurons in the same structures (as reported from striatum or hippocampal injections above) at levels comparable to that seen after injection of PFFs (Fig. 2). This suggests that the increased baseline levels of A53T α-syn protein in the transgenic mice (estimated at 1.3–2x protein compared to non-transgenic strain matched mice) ^20^ was sufficient to recruit the unfolded monomeric form of α-syn into the misfolded insoluble pSer129 α-syn.

**Figure 3:**
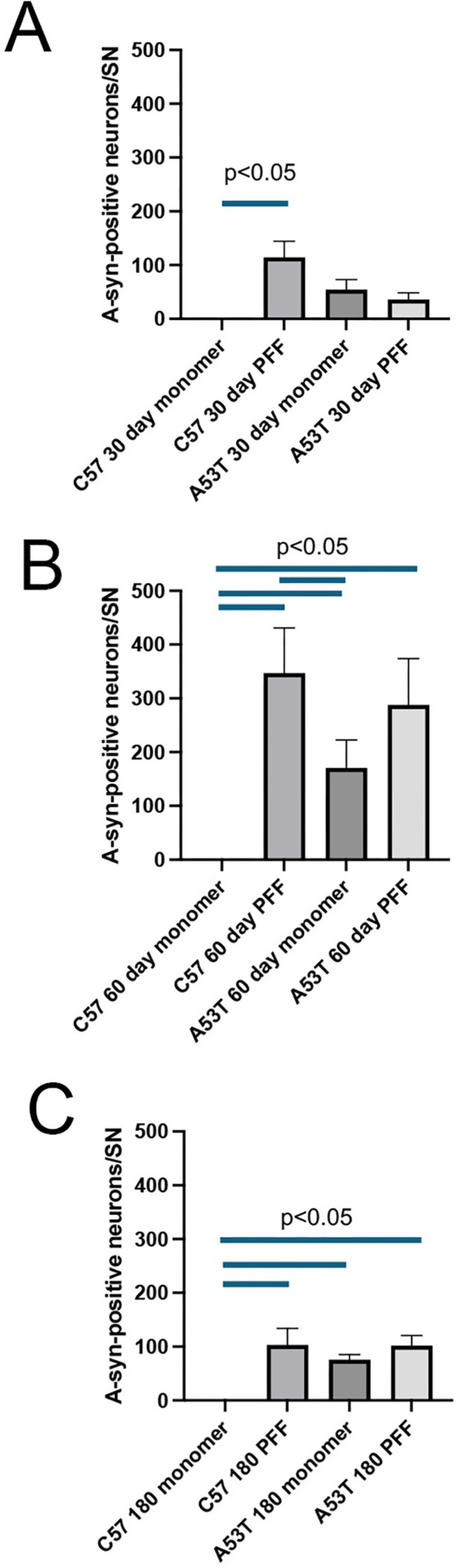
Quantitation of pSer129 α-syn-immunopositive SNpc DA neurons after injection of monomeric or PFFs into the striatum. A). 30 days after injection of monomeric α-syn into the striatum we did not find any pSer129 α-syn-immunopositive SNpc DA neurons. However, we did see a significant increase in the number of pSer129 α-syn-immunopositive SNpc DA neurons in mice injected with PFFs. Although we did detect pSer129 α-syn-immunopositive SNpc DA neurons in following monomeric or PFF injection of A53T mice, this increase was not significantly different from the C57BL/6J mice. B) 60 days following injection of monomeric α-syn we still do not observe any pSer129 α-syn-immunopositive SNpc DA neurons in the C57BL/6J mice. However, in A53T mice, injection of monomeric α-syn results in a significant increase in pSer129 α-syn-immunopositive SNpc DA neurons. In A53T mice, injection of either monomeric or PFFs induces a significant increase in the number of pSer129 α-syn-immunopositive SNpc DA neurons. The increase in pSer129 α-syn-immunopositive SNpc DA neurons at 60 days post PFF injection is significantly increased from 30 days in both the C57BL/6J (p<0.05) and A53T mice (p<0.01). C) 180 following injection of monomeric α-syn or PFFs, we still did not observe any pSer129 α-syn-immunopositive SNpc DA neurons after monomeric α-syn in the C57BL/6J mice. When PFFS are injected to C57BL/6J mice we still saw a significant increase in pSer129 α-syn-immunopositive SNpc DA neurons, although at this time we counted significantly lower numbers than at 60 days (p<0.05). In A53T mice, we measured a significant increase in the pSer129 α-syn-immunopositive SNpc DA neurons following both monomeric α-syn or PFFs compared to the C57BL/6 mice. However, at 180 days we also noted that the number of pSer129 α-syn-immunopositive SNpc DA neurons was significantly lower than at 60 days (p<0.05).

To further validate these qualitative observations, we used stereological methods to estimate the actual number of these pSer129 α-syn -positive DA neurons in the SNpc on the side of the monomeric or PFF injections (Fig. 1E) at 30, 60 or 180 days following injection of monomeric α-syn or PFFs into the striatum of C57BL/6J mice or A53T mice. In C57BL/6J mice, at either 30, 60 or 180 days post injection of monomeric α-syn we never observed pSer129 α-syn immunopositive SNpc DA neurons (Fig. 3,A-C). Thirty days following injection of PFF’s into the striatum of C57BL/6J mice, we counted an average of 115 +/− 30 pSer129 α-syn-positive DA neurons in the SNpc, which increased to 347 +/− 85 after 60 days. By 180 days post PFF injection, we counted 103 +/− 85 α-syn-positive SNpc DA neurons. We observed most of these pSer129 α-syn immunopositive DA neurons in the medial to caudal tier of the SNpc, although a few cells were present in each of the tier in the SNpc. Additionally, given that the number of SNpc DA neurons on each side of the mouse brain is approximately 6000 ^26,31,32^, our injection protocol only induced a-syn aggregation/transmission in, at most, 6% of the ipsilateral SNpc DA neurons,

When the number of α-syn-positive DA neurons were quantified in the SNpc of A53T (Fig. 3A-C). However, we did observe a significant difference between C57BL/6J and A53T mice following injection of oligomerized α-syn (PFFs), we noted a no significant difference in the number of neurons that were α-syn-positive compared to those measured in C57BL/6J mice following injection of monomeric α-syn. Unlike the C57BL/6J mice who never developed α-syn aggregates in the DA neurons of the SNpc, the A53T did show a significantly larger number of α-syn-positive SNpc DA neurons (55 +/− 18) at 30 days post-injection which increased to 171 +/− 51 α-syn-positive SNpc DA neurons 60 days post injection. This decreased to 75 +/− 9 α-syn-positive SNpc DA neurons at 180 days post injection (Fig. 3A-C).

#### Effect of PFF and monomeric α-Syn injection on SNpc DA neuron number

**Figure 4:**
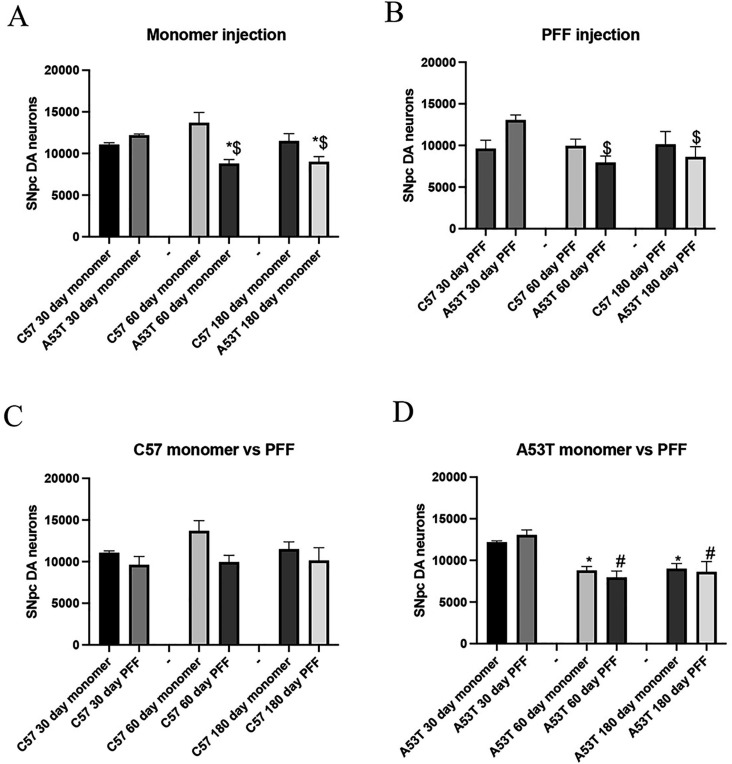
Effect of monomeric and PFF injection into striatum on SNpc DA neuron number. A). After monomeric α-syn injection, no loss of SNpc DA neurons were seen after 30, 60 or 180 days in C57BL/6J mice. However, in A53T mice, monomeric α-syn induced a significant loss of SNpc neurons at 60 and 180 days. B) After PFF injection, no loss of SNpc DA neurons were seen after 30, 60 or 180 days in C57BL/6J mice. However, in A53T mice, PFFs induced a significant loss of SNpc neurons at 60 and 180 days. * p<0.05 compared to same day monomer, $p<0.05 compared to 30 day timepoint C) Comparison of monomer versus PFFs in C57BL/6J mice. Neither monomeric α-syn nor PFFs induced a significant loss of SNpc neurons at 30, 60 or 180 days. D) Comparison of monomer versus PFFs in A53T mice. Neither monomeric α-syn nor PFFs induced a significant loss of SNpc neurons at 30 days post injection. However, at 60 and 180 days both the monomer and PFFs induced a significant and equal decrease in SNpc DA neuron number. * p<0.05 compared to 30 day monomer, # p<0.05 compared to 30 day PFF.

To further explore the effects of α-syn aggregation and transmission on SNpc DA neuron health, we performed a stereological evaluation to estimate the number of SNpc TH-positive DA neurons subsequent to monomeric or PFF injection to the striatum. In C57BL/6J mice, neither monomer nor PFF injection into the striatum (Fig. 4A,B,C) resulted in any loss of SNpc DA neurons. However, in A53T mice, there was significant SNpc TH+ (+ nissl) DA neuron loss at 60dpi and 180dpi in both PFF and monomer injected animals (Fig. 4A,B,D). These data, paired with the results from the α-syn immunohistochemistry, were striking for two reasons. One, we did not see SNpc TH + cell loss in the wild-type animals regardless of the form of α-syn injected. Secondly, we clearly demonstrate the ability of non-fibrillized α-syn monomer to both initiate aggregation and spread α-syn pathology, which we suggest induces a significant DA neuron loss in the SNpc. These results suggest that the presence of α-syn aggregates alone is not sufficient to drive neuron loss in the SNpc but may rely on the levels of induced neuroinflammation alone or in combination with α-syn burden.

### Effect of PFF injection on Immune Response in C57BL/6J and A53T mice.

**Figure 5:**
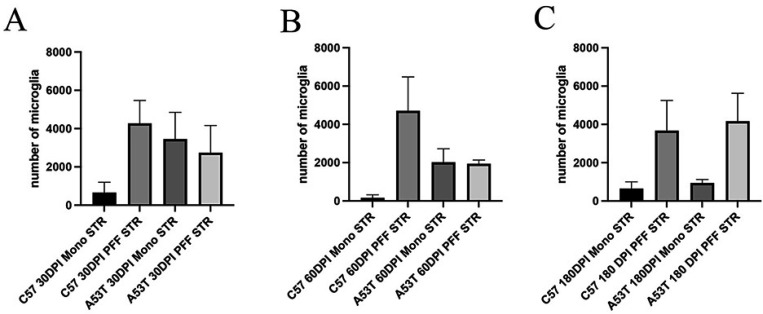
Stereological assessment of activated microglia in the SNpc following monomeric α-syn or PFF injection into striatum. A). 30 days after injection of monomeric or PFF α-syn we quantified a variable, but average 6–10 fold increase in the number of activated microglia in both C57BL/6J and A53T mice injected intrastriatally with PFFs. A similar increase was seen in A53T mice after monomeric α-syn. B) 60 days after injection of monomeric or PFF α-syn we quantified a variable, but average 6–10 fold increase in the number of activated microglia in both C57BL/6J and A53T mice injected intrastriatally with PFFs. C) 180 days after injection of monomeric of PFF α-syn we quantified a variable, but average 8–10 fold increase in the number of activated microglia only in mice injected intrastriatally with PFFs. The number of activated microglia in A53T injected with monomeric mice α-syn was similar to that seen in C57BL/6J mice.

To examine the immunological effects of intracerebral α-syn injection, we performed Iba-1 immunohistochemistry combined with stereological techniques to assess how exogenous α-syn affected the number and state of SNpc microglia in the SNpc. While PFFs induced an increase in activated microglia when compared to monomer injections, we observed important differences dependent on the form of α-syn as well as the recipient animal’s genotype. In C57BL/6J mice, injection of monomeric forms of α-syn did not induce any significant inflammatory effect in the SNpc at any of the timepoints examined (Fig. 5A,B,C). However, when PFFs were injected into dorsal striatum, and although extremely variable within groups, we measured an averaged 10-fold increase in the number of activated microglia at 30 dpi, which persisted through the 180 days of observation (Fig. 5C). A quite different picture was seen in A53T mice. At 30 dpi, monomeric α-syn induced a similar microglial activation to that seen after PFFs in C57BL/6J mice (Fig. 5A). The induction of microglia was similar in mice injected with PFFs at 30 dpi (Fig. 5A). At 60 days, we observed a progressive loss of activated microglia in animals injected with monomeric α-syn, so that by 180 dpi, they had returned to baseline numbers (Fig. 5C). In A53T mice injected with PFFs, the initial 30day increase in activated microglia was maintained for the entire 180-day period of observation.

**Figure 6:**
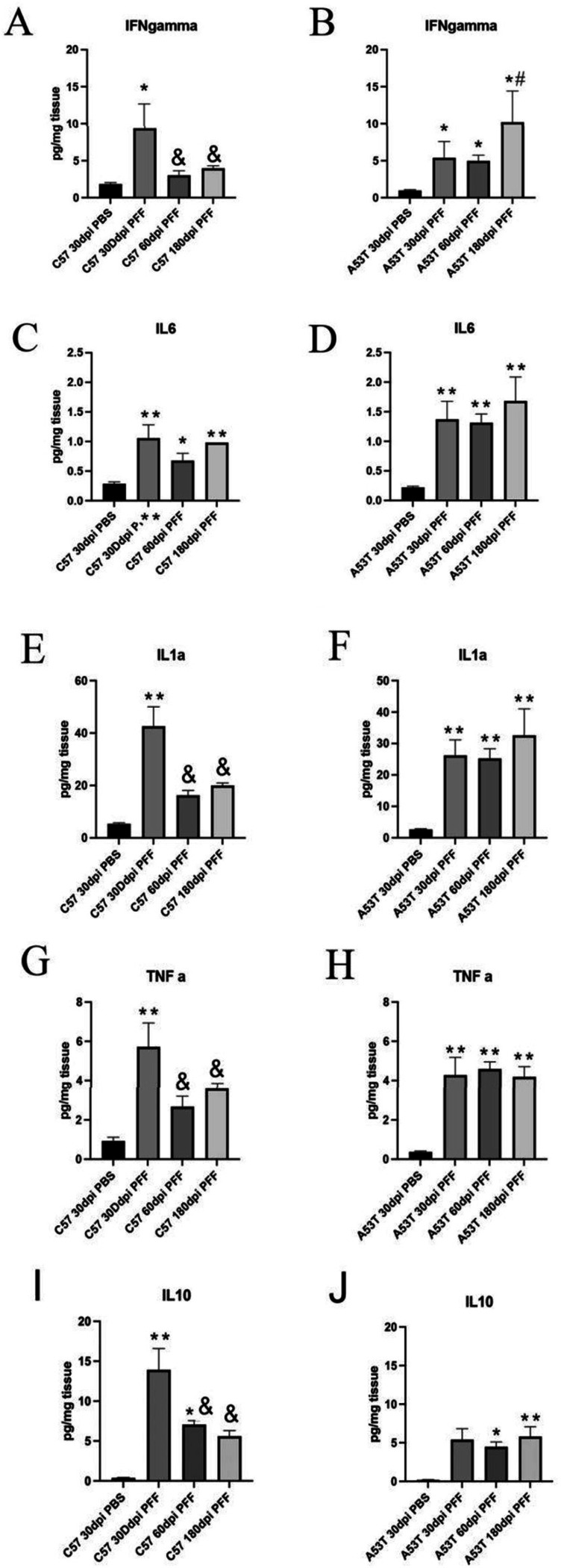
SNpc cytokine activation 30 days following intrastriatal injection of PFFs in C57BL/6J or A53T SNCA mice. A) Expression of IFNg was significantly increased 30 days after injection of PFFs in C57BL/6J mice compared to mice injected with PBS. At both 60 and 180 days after injection of PFFs, we see a significant increase in both C57BL/6J and mice compared to saline injected mice, but at this time, these levels have significantly been reduced compared to 30 days after PFFs. B) Expression of IFNg was significantly increased 30, 60 and 180 days after injection of PFFs in A53T SNCA mice compared to A53T SNCA mice injected with PBS. We also measured a significant increase in IFNgamma in AA53T SNCA mice compared to levels measured at 30 or 60 days after PFF injection. C) Expression of IL-6 was significantly increased 30, 60 and 180 days after injection of PFFs in C57BL/6J mice compared to C57BL/6J mice injected with PBS. D) Expression of IL-6 was significantly increased 30, 60 and 180 days after injection of PFFs in A53T SNCA mice compared to mice injected with PBS. E) Expression of IL1a was significantly increased 30, 60 and 180 days after injection of PFFs in C57BL/6J mice compared to C57BL/6J mice injected with PBS. F) Expression of IL1a was significantly increased 30, 60 and 180 days after injection of PFFs in A53T SNCA mice compared to A53T SNCA mice injected with PBS. G) Expression of TNFa was significantly increased 30, 60 and 180 days after injection of PFFs in C57BL/6J mice compared to C57BL/6J mice injected with PBS but at this time, TNFa levels have significantly been reduced compared to 30 days after PFFs. H) Expression of TNFa was significantly increased 30, 60 and 180 days after injection of PFFs in A53T SNCA mice compared to A53T SNCA mice injected with PBS. I) Expression of IL-10 was significantly increased 30, 60 and 180 days after injection of PFFs in C57BL/6J mice compared to C57BL/6J mice injected with PBS. J) Expression of IL-10 was significantly increased at 60dpi and 180dpi although it was 65% less than C57BL/6J mice. ** p<0.01 compared to

We also examined the expression of 4 proinflammatory cytokines (IFNg, TNFa, IL-6 and IL-1a) and one anti-inflammatory cytokine (IL-10) in the SNpc following intrastriatal injection of PFFs. In C57BL/6J mice, we measured a significant increase in each of the proinflammatory cytokines 30 days after PFF injection. By 60 dpi, IFNg, TNFa, and IL-1a had returned to baseline levels, while IL-6 levels remained elevated (Fig. 6A,C,E,G). Like the C57BL/6J mice, striatal PFF injection into A53T mice resulted in a significant induction of all 4 proinflammatory cytokines 30 dpi. However, unlike the C57BL/6J animals, the A53T mice demonstrated a continued elevation of these inflammatory mediators through the entire experimental period (180 dpi) (Fig, 6B,D,F,H). We also examined changes in the anti-inflammatory cytokine IL-10. C57BL/6J mice had significant increases at 30 and 60 dpi, which returned to baseline levels at 18o dpi (Fig. 6I). In A53T mice, we also measured a significant increase in IL-10 (Fig. 6J) but its induction compared to the C57BL/6J was only reduced by approximately 65% (Fig. 6I,J). Because of the risk of endotoxin induced neuroinflammation, we performed a TLR4 NF-kB reporter assay to evaluate endotoxin levels of our α-syn preparation. (Suppl Fig. 2). This assay demonstrated that our α-syn preparations (Proteos) had minimal activation of NF-kB suggesting that the endotoxin from α-syn is not responsible for the neuroinflammation observed.

**Figure 7:**
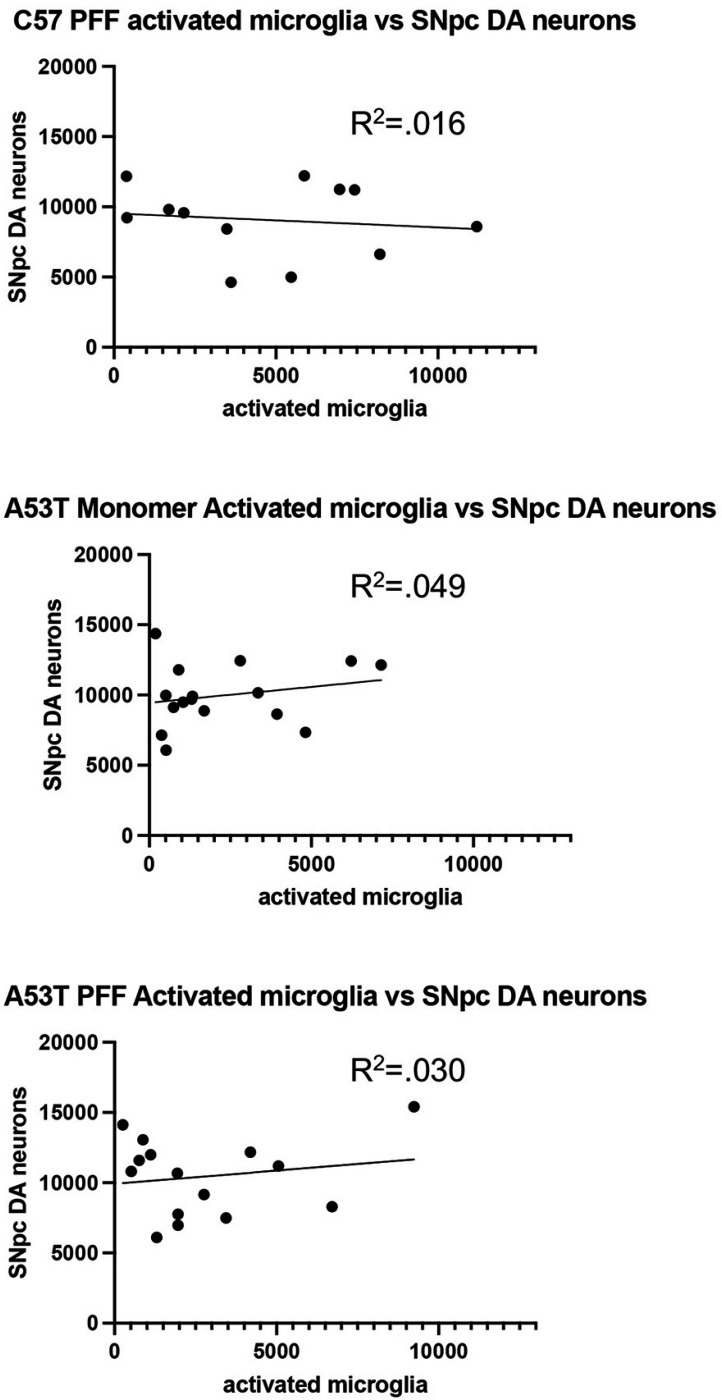
Correlation between activated microglia and SNpc DA neuron loss. No positive or negative correlation was measured in C57BL/6J PFF injected or A53T SNCA mice after monomeric or PFF injection into striatum. Each dot represents data from 1 animal.

Given the variability within groups of the number of activated microglia induced by monomer of PFF injection, we performed a regression analysis of individual animals to correlate the number of activated microglia with SNpc DA neuron loss. As seen in Fig. 7, we did not detect any significant correlation between these two variables. This suggested that any cell death seen after PFF injection in both C57BL/6J and A53T mice was likely due to direct synuclein burden. While we did not find any correlation between the number of activated microglia/animal and SNpc DA neuron death, we do find significant increases in proinflammatory cytokines following injection of PFFs. This suggests that these chemical mediators, not the number of activated microglia, better serve in this model as the inflammatory contributor to the observed pathology.

## Discussion

Our findings in this study, using a PFF model of induced synucleinopathy, support previous work that has demonstrated the potential for specific transmission of fibrillar α-syn fragments ^22,23,33,34^. We also show that the transmission of these fragments is not only permissive in the nigrostriatal tract but can also occur in other synaptically-connected regions. Additionally, we also show that monomeric forms of α-syn can be recruited into insoluble aggregates in brains when the endogenous α-syn burden is already at higher than physiological levels. This observation may provide insight that provides a mechanism for the early onset of parkinsonism seen in humans that carry duplication/triplication, without any mutation, of the SNCA gene ^35,36^.

While the presence of insoluble α-syn inclusions in the SNpc- and throughout the neuroaxis- are hallmarks of PD, there is active debate about the correlation between α-syn burden and degree of neuronal dysfunction. Studies in animals offer evidence that formation of inclusions coincides with neuronal dysfunction, while others show a weak correlation between inclusion burden and loss of SNpc TH neurons after striatal PFF injections in rodent models. ^37,38^. However, in post-mortem tissue of PD patients, the relationship between protein load and SN degeneration is unclear, with some patients experiencing PD symptoms and loss of nigral neurons without α-syn aggregates ^39–42^. Additionally, Lewy pathology is present in other neurodegenerative diseases that are clinically different from PD ^43^. While the relationship between the spread of Lewy pathology and clinical progression of PD has been outlined by Braak and his colleagues ^13,44^, only about half of people with clinical PD have a distribution of Lewy pathology that exhibits the classic pattern of Braak staging, and those with genetic forms of disease can be even more distinct from this pattern of synuclein deposition ^45^. Critical to our understanding of α-syn spread is the finding that Lewy pathology is hypothesized to spread through synaptically-connected networks of neurons, rather than through its nearest neighbor ^23,46^. While this hypothesis has clear support from our injections to striatum (progression in the nigrostriatal system) and hippocampus (progression in the limbic system), there are reports that some regions of high Lewy pathology burden, such as locus coeruleus, do not act as nodes of propagation ^47,48^. The heterogeneity in PD and related synucleinopathies between Lewy body formation and neurodegeneration suggests there are distinct regional brain differences that could be governing this relationship. It has been shown that there are differences within the midbrain (medial vs. ventrolateral SN, and SN vs. ventral tegmental area (VTA) regarding axonal arbor dimension, pacemaking mechanisms, calcium signaling, and mitochondrial oxidative stress that results in various levels of cell vulnerability in neurodegeneration ^49^. With such variability in the effects of Lewy pathology in various brain regions, we focused on characterizing inflammation which could be a crucial factor in understanding the cellular context of neurodegeneration in synucleinopathies.

Regarding our examination of inflammation secondary to injection of monomeric or oligomerized α-syn, we found no significant correlation between the number of activated microglia in the SNpc and the loss of SNpc DA neurons. However, we did find that when SNpc DA neurons were lost there was a significant increase in the expression of proinflammatory cytokines in this tissue. This experimental finding is supported by the observation, in humans with PD, of microglia that immunostain for the pro-inflammatory cytokines TNFa and IL-6 ^50^. This suggests, at least following induction from overexpression of α-syn, that the morphological appearance of these inflammatory cells alone, cannot be used as a marker of toxic inflammation, but should be used in conjunction with direct measurement of their inflammatory secretome. Additionally, and while there is no conclusive evidence from our studies, it does seem that the increase in proinflammatory cytokines does temporally precede SNpc DA neuron death. This suggests to us that both inflammation combined with increased α-syn burden is required for the death observed in SNpc DA neurons ^51–54^. The role of PFFs in driving this inflammation may also be related to the brain’s overall synuclein burden. For example, we do not see any rise in microglial number-nor cytokine expression- in C57BL/6J mice injected with monomeric forms of α-syn; but do see this in A53T mice. While it has been shown that the mutant A53T form of α-syn is capable of more strongly activating wild-type microglia in mice, it was previously unknown how mutant A53T microglia are able to respond to exogenous wild-type human α-syn ^53^. While we do not know if the form of synuclein drives inflammation or if the protein must first template into oligomeric forms, it has been reported that α-syn monomer does not activate microglia in vitro ^55^. To ensure that our any of our immunopathology was secondary to exogenous α-syn we performed a NF-kB reporter assay to quantify any endotoxin that potentially contaminated our preparations, and confirmed that our findings were not due to this potential artifact-inducing protein.

The mutant mice we used in this study overexpressed human A53T synuclein on a mouse SNCA null background. This transgenic PAC induced a 1.3–2 fold overexpression of human α-syn protein (8–21-fold increase in mRNA) compared to the typical exogenous mouse α-syn levels ^20^. It is possible that this overexpression of soluble a-synuclein, which is similar to that seen in humans with duplication of the SNCA gene ^56^ puts stress on the microglial load for handling protein degradation, such that exogenous application induced an overall pro-inflammatory response. If this is the case, then two possible deleterious consequences could arise: 1) microglia have reached maximum capacity for protein degradation and cannot maintain protein homeostasis. Mechanistically, this would occur via a gain of inflammatory phenotype or loss of anti-inflammatory phenotype, furthering cellular stress and protein propagation, or 2) the microglial activity is altered, potentially leading to the development of more toxic species of α-syn oligomers that subsequently drive propagation and inflammation. Additionally, we know that monomeric α-syn can interact with aggregated α-syn via intramolecular unfolding, which promotes further protein aggregation ^57^. While it has not been previously reported in the A53T double PAC mutant mice, M83 A53T mice do report increasing levels of oligomeric α-syn with age, with the A53T double PAC mice having some evidence of aggregated α-syn in the ENS and dystrophic neurites in the hippocampus ^20,58^. Since there may be increased levels of aggregated α-syn in the A53T double PAC mice, it is possible that intrastriatal monomeric injections of α-syn are capable of directly interacting with oligomeric species to accelerate pathology. Furthermore, not only is the proteomic environment permissive of aggregation, but also the transgenic immune environment, namely microglia, could contribute to the aggregation process. It is important to note that our findings conflict with a recent study showing no aggregation when utilizing control brain homogenate in A53T animals ^59^. These two findings could be copacetic as we utilize purified α-syn where the other group uses whole brain homogenate, which could prove to be a determinant factor in the initiation of aggregation. Finally, it should be noted that we initially designed these experiments utilizing human α-syn as the injection material seeking to synergize with the A53T animals to produce maximum pathology since sequence homology has been shown to dictate seeding efficiency ^25^. While it is possible that this species barrier could have resulted in reduced pathogenicity in the C57BL/6J animals injected with PFFs, it does not account for the differences in pathogenicity of α-syn monomeric injections. For these reasons, the handling and processing of α-syn by microglia must be central to future studies.

## Conclusions

Our results reinforce the pivotal role that microglia play in homeostasis of the nigrostriatal pathway. Here, we demonstrate that in mice overexpressing A53T α-syn, any exogenous application of α-syn, whether monomeric or fibrillized, is capable of inducing an inflammatory cascade and suppressing anti-inflammatory signaling. We also find that the inflammatory secretome play a central role both in the response to α-syn aggregation as well as in mediating pro- and anti-inflammatory responses in a PFF model of synucleinopathy. In terms of disease progression, our finding of monomeric induction of a parkinsonian pathology in mice that overexpress the A53T form of this mutation- which is the WT form in mice ^60^ may provide a mechanism to explain the earlier onset of disease in persons carrying duplication/triplication of the WT SNCA gene. This may also point to a mechanism underlying disease initiation and progression of other proteinopathies, such as those arising from tau, that that arise from overexpression of normal unmutated proteins.

## Supplementary Material

Supplement 1

## Data Availability

The datasets used and/or analyzed during the current study are available from the corresponding author on reasonable request.

## Abbreviations

α-syn: alpha synuclein
CNS: central nervous system
DA: Dopamine
DMV: dorsal motor nucleus of the vagus nerve
HPLC: High performance liquid chromatography
HC: Histochemistry
IHC: immunohistochemistry
PAC: P1-derived artificial chromosome
PD: Parkinson’s disease
p129Ser: phosphorylation of alpha synuclein at serine129
PFFs: pre-formed fibrils of alpha-synuclein
SNpc: substantia nigra pars compacta
TH: tyrosine hydroxylase
VTA: ventral tegmental area
WT: wild-type
