# Supplementary material for: Templating of Monomeric Alpha-Synuclein Induces Inflammation and SNpc Dopamine Neuron Death in a Genetic Mouse Model of Synucleinopathy
Source: Res Sq. 2024 Nov 20:rs.3.rs-5269499. Preprint. [Version 1] doi: 10.21203/rs.3.rs-5269499/v1 (PMC11601858; doi:10.21203/rs.3.rs-5269499/v1)
Supplement: Supplement 1 [file NIHPPRS5269499V1-supplement-1.pdf]

Supplementary Files

This is a list of supplementary files associated with this preprint. Click to download.

- SupplFigures8824.pdf
